# Investigating the association of proton pump inhibitors with chronic kidney disease and its impact on clinical practice and future research: a review

**DOI:** 10.1186/s40545-019-0167-0

**Published:** 2019-03-29

**Authors:** Ejaz Cheema

**Affiliations:** Institute of clinical sciences, University of Birmingham, Edgbaston, Birmingham, B15 2TT UK

**Keywords:** Proton pump inhibitors, Chronic kidney disease, Clinical practice

## Abstract

**Background:**

Proton pump inhibitors (PPIs) are used worldwide for the treatment of gastroesophageal reflux disease (GERD) and peptic ulcer disease (PUD). Although considered to be widely safe, PPIs have been associated with the potential risk of adverse effects such as infections including pneumonia and *Clostridium difficile*, malabsorption of vitamins and minerals, dementia and more recently with chronic kidney disease (CKD). Evidence including large cohort studies suggests that there is a greater risk of developing CKD in chronic users of PPIs. However, the association of CKD with PPI use reported in these studies is weak and does not establish a clear causality. This review aims to further investigate the association of CKD with PPI use by including studies with various study designs.

**Methods:**

A literature search of published articles with no start date restrictions was undertaken in May 2018 in three electronic databases (PubMed, ScienceDirect, Google Scholar). Search terms included ‘Proton Pump Inhibitors’, ‘chronic kidney disease’, and ‘association’. Both observational and randomised controlled trials (RCTs) investigating the association of CKD with PPI use were eligible for inclusion.

**Results:**

Ten observational studies with 1,005,899 patients contributed to the review. No experimental study was available for inclusion in the review. Of the included studies, six used a retrospective study design, while the rest were prospective (two) or a case-controlled studies (two). A large prospective cohort study with 144,032 patients conducted in the USA reported that PPI use compared to no PPI use was associated with an increased risk of CKD Hazard ratio [HR] 1.28; 95% Confidence Interval [CI] 1.22–1.34. However, the observational study design of this study together with other studies included in the review suggests that the strength of evidence associating PPI use with CKD is weak and does not establish true causality.

**Conclusions:**

The current evidence related to the potential association of CKD with PPI use remains inconclusive in establishing true causality. Further prospective studies including randomised controlled trials and cohort studies would be required to confirm the findings reported in this review and to draw any conclusions.

## Background

Proton pump inhibitors (PPIs) are used worldwide for the treatment of gastroesophageal reflux disease (GERD) and peptic ulcer disease (PUD) [1, 2]. For example, evidence from the National Health and Nutrition Examination Survey that was conducted to assess the use of prescription drugs among adult United States citizens reported that around 8% of the population had taken a PPI in the preceding one month [3]. It is important to highlight that these findings do not represent the non-prescription use of PPIs that are also available over the counter (OTC) in the United States (US) [4]. Such widespread use of PPIs has also been reported in the United Kingdom (UK) [5]. This study not only suggested an increase in the public use of PPIs but also reported an increased prevalence of chronic users of PPIs in the UK [5].

Although considered to be widely safe, PPIs have been associated with the potential risk of adverse effects such as infections including pneumonia and *Clostridium difficile*, malabsorption of vitamins and minerals, dementia and more recently with chronic kidney disease (CKD) [6]. CKD which has a sustained global prevalence of 11 to 13%, is among the most significant long-term medical conditions in the world [7]. It is a known risk factor for cardiovascular disease and its associated mortality and morbidity. Evidence including large cohort studies suggests that there is a greater risk of developing CKD in chronic users of PPIs [8–10]. However, the association of CKD with PPI use reported in these studies is weak and does not establish a clear causality. Although, a recently conducted meta-analysis involving five studies with 536,902 participants also suggested a greater risk of CKD in PPI users compared to the users of Histamine 2 receptor antagonists (H2RA), the review was limited with the inclusion of observational studies only [11]. Furthermore, this review did not discuss the implications of the findings on clinical practice. The current narrative review therefore aims to further investigate the association of CKD with PPI use by including studies with various study designs and to discuss the implications of the findings on both clinical practice and future research.

## Methods

### Search strategy for identification of studies

A literature search of published articles with no start date restrictions was undertaken in May, 2018 in three electronic databases (PubMed, ScienceDirect, Google Scholar). Search terms included ‘Proton Pump Inhibitors’, ‘chronic kidney disease’, and ‘association’. Both observational and randomised controlled trials (RCTs) investigating the association of CKD with PPI use were eligible for inclusion. A narrative review of the included studies was undertaken.

## Results

### Studies reporting association of CKD with PPI use

Ten observational studies contributed to the review (see Table 1 for summary of characteristics of included studies). A retrospective study including 1284 patients was conducted in Korea to investigate the association of duration of PPI use with CKD outcomes including incident CKD, incident CKD with progression and mild renal progression [12]. Using Cox regression model, the study reported no significant association between duration of PPI use and chronic kidney disease (Hazard Ratio of heavy users, 1.50; 95% CI, 0.61–3.67). However, the study did report that prolonged use of PPI was associated with mild renal progression in patients who were younger than 65 years (HR of heavy users, 2.24; 95% CI, 1.09–4.60). It is important to highlight that this study included patients with coronary artery disease that made them more susceptible to the risk of developing renal complications due to their cardiovascular comorbidities. This study was therefore limited in generalising its finding to the wider population.

**Table 1:** Summary of studies included in the review

Author, year of study	Country	Study design	Number of participants	Outcomes assessed	Results
Cho et al., 2018 [12]	Korea	Retrospective study	1284	CKD outcomes (incident CKD, incident CKD with progression and mild renal progression)	No significant association between duration of PPI use and chronic kidney disease development (HR of heavy users, 1.50; 95% CI, 0.61–3.67),
Rodriguez-Poncelas et al., 2018 [15]	Spain	Population-based retrospective study	46,541	Incident CKD	Higher doses of PPIs increased the risk of incident CKD (HR 1.92; 95% CI 1.00–6.19).
Hung et al., 2018 [13]	Taiwan	Population-based case-controlled study	16,704	Incidence of CKD	Odds Ratio for CKD in PPI users was 1.41 compared to non-users of PPI
Klatte et al., 2017 [14]	Sweden	Retrospective study	114, 883	Progression of CKD (estimated by doubling of creatinine levels and reduction in glomerular filtration rate	PPI use associated with raised creatine levels compared to HR receptor antagonists (1985 events; adjusted hazard ratio [HR], 1.26; 95% CI, 1.05–1.51) as well as reduction in the estimated GFR by 30% or more (11,045 events; 1.26; 95% CI, 1.16–1.36).
Lazarus et al., 2016 [8]	USA	Prospective cohort study	10,482	Incidence of CKD	Risk of CKD with PPI use was 1.39 (1.01–1.91) against no PPI use
Arora et al., 2016 [9]	USA	Retrospective study	99,269	Incidence of CKD	PPI use against no PPI use 1.10 (1.04–1.16)
Xie et al., 2016 [10]	USA	Prospective cohort study	144,032	Incidence of CKD	The risk of CKD associated with PPI was 1.28 (1.22–1.34) against no PPI use
Blank et al., 2014 [20]	New Zealand	Nested case-controlled study	572,661	Incidence of PPI-induced AIN	The incidence rates and confidence intervals per 100,000 person-years were 11.98 (9.11–15.47) for current and 1.68 (0.91–2.86) for past users of PPIs
Simpson et al., 2006 [19]	New Zealand	Observational study (Reports and analysis)	15	Incidence of PPI-induced AIN	The incidence of AIN was found to be in 8 per 100 000 patient years (95% confidence level 2.6–18.7)
Geevasinga et al., 2006 [18]	Australia	Retrospective case review	28	Incidence of PPI-induced AIN	The study detected 18 cases of biopsy-proven PPI-induced AIN

Another study that reported a weaker association of PPI use with CKD was conducted in Taiwan [13]. This large case-controlled study included 16,704 patients aged 20 or above and had been diagnosed with CKD. The study was aimed to investigate the association of CKD with ‘PPI use’ defined as patients who had received one prescription of PPI prior to the start of the study. Using logistic regression model, the study reported an Odds Ratio (OR) of 1.41 for CKD in patients who had used PPI [95% confidence interval (CI) 1.34–1.48] compared to patients who had never used PPIs before. Furthermore, the weaker association of CKD with PPIs was not limited to one PPI but was extended to all types of PPIs.

A study that did report relatively stronger association of PPI use with CKD was conducted in Sweden [14]. This retrospective study included 105,305 users of PPIs and 9578 users of H2 receptor antagonists. The study was aimed to investigate the association of PPI use with the progression of CKD defined as ‘two-fold increase in creatinine levels or 30% reduction in estimated glomerular filtration rate (GFR). The study reported that PPI users compared to HR receptor antagonists had both greater risk of raised creatine levels (1985 events; adjusted hazard ratio [HR], 1.26; 95% CI, 1.05–1.51) as well as reduction in the estimated GFR by 30% or more (11,045 events; 1.26; 95% CI, 1.16–1.36). Furthermore, PPI use was also reported to be linked with the risk of end-stage kidney disease and acute kidney injury. However, the findings of this study should be confirmed in further studies including RCTs to establish true causality between PPI use and incidence of CKD.

Other observational studies that reported association of CKD with PPI use included a large prospective cohort study that involved 10,482 adults and was conducted to investigate the incidence of CKD with the use of PPIs among US population [8]. Participants were followed for a period of 14 years. The study included participants who received an outpatient prescription of PPI or self-reported using a PPI and were compared with participants who were using H2 receptor antagonists. The study reported that PPI users had a 20–50% higher incidence of CKD as compared to users of H2 receptor antagonists. Furthermore, the study also suggested that participants receiving a twice daily dose of a PPI had a greater risk of developing CKD as compared to users of both once daily regimen of PPI or users of H2 receptor antagonists [8].

Similar observations were reported in two other American studies that were aimed to determine the association of CKD with PPI use [9, 10]. The first study included 99,269 patients and by using a retrospective design reported that a quarter (25%) of the study patients who developed CKD during the study were taking PPIs [9]. Patients taking PPIs had a greater risk as compared to those without PPIs. The second study used a large cohort of 144,032 participants and was conducted to assess the impact of long-term PPI use on renal outcomes [10]. The study included 125,596 users of PPIs and 18,436 users of H2 receptor blockers. Participants were followed up for a period of 5 years. At the 5 year follow up, participants using PPIs were found to have a greater incidence of developing long-term renal outcomes as opposed to H2 receptor blockers [10]. Higher doses of PPIs together with their longer duration of use were reported to be associated with a greater risk of CKD [15]. A population-based retrospective cohort study conducted in Spain that involved 46,541 participants suggested that high doses of PPIs augmented the risk of incident CKD (HR 1.92; 95% CI 1.00–6.19). Furthermore, the reported risk of CKD increased after three to six months of exposure to PPIs (HR1.78; 95% CI 1.39–2.25).

One of the factors that can possibly explain the potential association between PPI use and CKD is the development of acute interstitial nephritis (AIN) in PPI users [9, 16]. Drug-induced AIN is responsible for majority of the cases with most patients presenting with an acute deterioration of renal function leading to the need for dialysis. Evidence suggests that around 30–70% of patients affected by the drug-induced AIN fail to return to their baseline renal function probably due to the swift conversion of interstitial cellular infiltrates into large areas of fibrosis [17]. It was evident from the findings of an Australian study that used a retrospective study design to determine the incidence of PPI-induced AIN [18]. The study detected 18 cases of biopsy-proven PPI-induced AIN that led to the development of acute kidney injury (AIN). Although all patients affected with PPI-induced AIN improved their renal function, yet their mean calculated creatinine clearance was reported to be 15.9 ml/min/ 1.73 m2 and 11.5 ml/min/1.73 m2 that remained lower than baseline at three and six months respectively [18]. An observational study conducted in Auckland, New Zealand that was aimed to assess reports of patients diagnosed with AIN and renal failure from PPI use over a period of three years reported AIN incidence of 8 per 100,000 patient years (95% confidence level 2.6–18.7) [19]. Similar findings were reported in another Kiwi study that assessed the incidence of AIN in current and past users of PPIs [20]. This nested case-controlled study with 572,661 patients reported incidence rates and confidence intervals per 100,000 person-years as 11.98 (9.11–15.47) for current and 1.68 (0.91–2.86) for past users of PPIs. These findings suggested, that compared to past use, the current use of PPIs was linked to a significant risk of AIN.

## Discussion

The evidence presented in this review is limited to observational studies and does not include any experimental studies. The retrospective design of some of the included studies in this review together with the risk of selection bias and confounding factors suggests that findings of this review should be interpreted with caution. The strength of association reported in the included studies is also critical in determining causality between CKD and PPI use. Hypotheses generated from false associations can become a potential source of discontinuation of an established drug treatment. For example, two studies [12, 13] included in the review only reported a weak association of CKD with the PPI use. Both weak as well as strong associations could be explained by the risk of confounding [21] and thus have limited merit in establishing causality when compared to RCTs. The associations reported in this review would therefore need to be confirmed in further prospective studies including RCTs and cohort studies to evaluate the clinical outcomes and to draw any conclusions. RCTs that have a planned mechanism to control any known or unknown confounders should be the goal of future research.

Despite the study limitations of observational studies, the presumed weak or strong association of CKD with PPI use reported in this review may be well enough to raise unnecessary concern among both users and prescribers of PPIs. In view of these findings, there is a need to take a more pragmatic and cautious approach is required towards the prescribing of PPIs. For example, patients using PPIs for conditions where they are considered the drug of choice (GERD, PUD) [1, 2] should continue to use them in the lowest effective dose and for shortest possible time unless indicated otherwise. Special considerations should be given to elderly patients with multiple comorbidities and drug treatments at the time of initiating treatment with PPIs. Furthermore, given the availability of PPIs as OTC drugs worldwide, there is a need to educate patients about the unlicensed indications of PPIs and its associated adverse effects to curtail the overuse of PPIs.

## Conclusions

The current evidence related to the potential association of CKD with PPI use remains inconclusive in establishing true causality and should therefore not lead to unwarranted discontinuation of PPIs against established medical conditions such as GERD and PUD. While it is necessary to conduct further high-quality research including RCTs to determine causal inference, a more sensible and cautious approach should be adopted in the meantime by both users and prescribers to curtail the overuse of PPIs and its associated adverse effects.
